# Microglia as modulators of exosomal alpha-synuclein transmission

**DOI:** 10.1038/s41419-019-1404-9

**Published:** 2019-02-20

**Authors:** Yun Xia, Guoxin Zhang, Chao Han, Kai Ma, Xingfang Guo, Fang Wan, Liang Kou, Sijia Yin, Ling Liu, Jinsha Huang, Nian Xiong, Tao Wang

**Affiliations:** 1 Department of Neurology, Union Hospital, Tongji Medical College, Huazhong University of Science and Technology, Wuhan, China; 2 Department of Neurology, the First Affiliated Hospital of USTC, Division of Life Sciences and Medicine, University of Science and Technology of China, Hefei, China

## Abstract

Recent researches regarding to exosomal involvement in alpha-synuclein (α-syn) transmission relating to the pathological process of Parkinson’s disease (PD) have attracted considerable attention. It is highly desirable to make clear the diffusion process and cellular uptake of α-syn-associated exosomes and the underlying mechanism of exosomes-involved communication in the synucleinopathy pathogenesis. To determine the contribution of α-syn-associated exosomes to the initiation and progression of PD, plasma exosomes derived from PD patients were stereotaxically injected into the striatum of mice brains. Exosomes extracted from plasma diagnosed with PD contained monomeric and oligomeric α-syn. Here, we found that microglia display a high potency for uptake of plasma exosomes derived from PD patients, and therefore could be activated by exogenous exosomes in vitro and in vivo. In addition, immunofluorescent double staining verified the transfer of exogenous human exosomal α-syn to neurons. The release of human exosomal α-syn from microglia may facilitate this propagation. Finally, we described a mechanism underlying this potential role of microglia in the transmission of exosomal α-syn. Specifically, exogenous exosomes were found to dysregulate autophagy of the BV2 mouse microglia cell line with presentation of increased accumulation of intracellular α-syn and accelerated secretion of α-syn into extracellular space. These results suggest that microglia play a crucial role in the transmission of α-syn via exosomal pathways, in additional to idea that the progression of PD may be altered by the modulation of exosome secretion and/or microglial states.

## Introduction

Parkinson’s disease (PD) is a neurodegenerative movement disorder that is neuropathologically characterized by the accumulation of intraneuronal alpha-synuclein (α-syn)^1,2^. In addition, a growing body of evidence from animal models, as well as data from cultured cells and human postmortem brains, support the idea that α-syn can propagate from cell to cell, suggesting an important role of extracellular α-syn in its aggregation^3–6^. Several mechanisms related to the cell-to-cell transmission of α-syn have been reported, including the involvement of exosomes, tunneling nanotubes, classical exocytosis and endocytosis, trans-synaptic junctions, and direct penetration^7–12^. Recently, α-syn is identified within exosomes of human biological fluids, such as blood plasma and cerebrospinal fluid (CSF), as well as within the conditioned media of neuronal cells. This implies exosomes as the essential carriers for intercellular α-syn transmission^7,13–15^. Interestingly, α-syn is detected both inside the exosomes and on their membrane surface^10^. Notably, variations of plasma or CSF exosomal α-syn protein levels have been found to be associated with the occurrence of synucleinopathies^13,15^. However, little attention has been devoted to the species of exosomal α-syn. Data from published literatures in regard to this issue suggests that α-syn oligomers, but not monomers or fibrils, may represent the most neurotoxic species by presumably targeting, in vitro and in vivo, the formation of α-syn pathological aggregation^16–18^. Similarly, some recent studies demonstrated that α-syn aggregates could be induced by the introduction of exosomal α-syn derived from patients with synucleinopathies into cultured cells or wild-type mice^15,19^. It is surely meaningful to explore the potential relevance between the levels of exosomal oligomeric α-syn and the progression of PD.

Exosomes, a subset of extracellular vesicles, have recently been detected to penetrate the blood–brain barrier and thus may result in the spread of brain protein to the plasma pool^20–22^. Interestingly, recent experiments also indicate that central nervous system (CNS) derived α-syn may efflux from the brain to the plasma by exosomes, so, plasma exosomal α-syn may reflect the CNS status^13^. In addition, exosomal secretions are a crucial mean of clearing pathological proteins, including α-syn^23–25^. The intracellular accumulation of α-syn has been associated with the dysfunction of mitochondrial metabolism, ubiquitin-proteasome system, autophagy–lysosomal pathway, endoplasmic reticulum stress, and calcium homeostasis^26^. Increasing evidence has suggested that inhibition of autophagy can result in the formation of fused autophagosome–multivesicular bodies compartment and the release of exosomal α-syn^27,28^. Therefore, an imbalance between these pathways may lead to abnormal secretion of α-syn-carrying exosomes that might contribute the propagation of α-syn, and the following disease progression.

Microglia, the resident macrophages with secretory properties within the CNS, display a high capacity for elimination of extracellular α-syn, suggesting an important role of microglia in the modulation of synucleinopathies^29–31^. Excessively pathological α-syn can be taken up by surrounding microglia, which facilitates neuroinflammation and the following neurodegenerative events by releasing inflammatory mediators^32–34^. However, the cause of the microglia-mediated dopaminergic neurotoxicity is not yet completely clear. The protein cargo of exosomes from activated microglia may be involved. It is worth noting that, in contrast to neurons and astrocytes, microglial cells have been found to be targets of exogenous exosomes in cellular and animal models of Alzheimer’s disease^35–37^. A recent study has described the microglial contribution to the propagation of tau via exosomal pathways in vitro and in vivo^38^. Furthermore, elimination of microglia and the inhibition of exosome synthesis prevented the transmission of tau pathology^38,39^. Take the similarity of α-syn and tau into consideration, we investigated the role of microglial cells in the transmission of α-syn-containing exosomes, and vice versa the role of exosomes in affecting microglia and promoting its further spread, and offer a new insight into the mechanism of exosomes in PD pathogenesis.

## Materials and methods

### Participants

Blood samples used in this study were collected from Union Hospital, Tongji Medical College, Huazhong University of Science and Technology, following approval from the ethics committee. In this study, 20 PD patients (mean age 70.0 ± 6.22 years) and 15 age-matched healthy controls (mean age 68.2 ± 3.12 years) were included. All patients underwent a professional clinical assessment by three neurologists. A diagnosis was established according to the 2015 Movement Disorder Society clinical diagnosis criteria^40^. Furthermore, brain magnetic resonance imaging, blood biochemistry, and other laboratory tests, were performed to exclude secondary Parkinsonism. The criteria for enrollment of PD patients were: (1) age over 60 years; (2) disease duration over 5 years; and (3) PD drug naivety. Patients with idiopathic PD were clinically evaluated for PD severity using the Unified Parkinson’s Disease Rating Scale (UPDRS) part III and Hoehn & Yahr scale. Healthy controls should be age-matched and had a negative-disease history. No participant had a history of cancer, cerebrovascular disease, chronic infectious disease, and genetic disorders. All the participants were informed about the study and agreed to participate by signing an informed consent form.

### Exosomes isolation, characterization, and labeling

Exosomes were isolated from human plasma and conditioned media using differential centrifugation and ultracentrifugation following a protocol adapted from Théry et al.^41^. Briefly, the sample was collected and spun at 2000×*g* for 30 min followed by 12,000×*g* for 45 min. The supernatant were transferred to ultracentrifuge tube and centrifuged at 110,000×*g* for 120 min. The pellet was diluted with phosphate-buffered saline (PBS) and filtered through a 0.22-μm filter. The sample was then transferred to a clean tube and centrifuged again at 110,000×*g* for 70 min. The exosomes-containing pellet was resuspended in 50–100 μl of PBS or radio-immunoprecipitation assay (RIPA) lysis buffer for further experiments. All centrifugations were performed at 4 °C.

The size and morphological characteristic of the exosome preparations were analyzed using electron microscopy (EM). A drop of exosomes was fixed with 1% glutaraldehyde for 5 min, applied to a Formvar-carbon coated EM grid, and contrasted in 2% phosphotungstic acid. Then exosomes were observed under the electron microscope at 80 kV.

Purified exosomes were labeled with the red fluorescent lipophilic linker PKH26 or PKH67 (Sigma) following the manufacturer’s instructions. Briefly, 100 μl of exosomes was diluted in 1 ml Dilution C. Separately, 4 μl of PKH was mixed with 1 ml Dilution C. Then exosome suspension was added to the mixture followed by incubation at room temperature for 4 min. The stain reaction was stopped with 2 ml of 1% bovine serum albumin. After incubation, PKH-labeled exosomes were pelleted by ultracentrifugation.

### Super-resolution imaging and light-sheet live cell imaging of plasma-derived exosomes

Dilute exosomes-containing solution at 1:20 dilution with PBS. Add α-syn antibody (Abcam, ab138501) to solution to get 1:200 dilution, and incubate at room temperature on a PLL-coated coverslip for 90 min. Wash with PBS three times. Add Alexa Fluor 750 labeled goat anti-rabbit IgG at 1:1000 dilution, and incubate at room temperature on a PLL-coated coverslip for 90 min. Stain with 0.1 M DiD (a flurescent lipophilic dye) for 5 min. Wash with PBS three times. Observations were made with super-resolution microscopy.

We placed 8 mm-diameter coverslips inside a 12-well plate, and seeded human microglia (HM) (ScienCell, Human Microglia, Catalog # 1900) cells into the wells. On the following day, we made a freshly prepared medium containing with 2 μM SiR-tubulin (Cytoskeleton, Catalog # CY-SC002), removed the coverslips and placed them inside the freshly prepared medium. Subsequently they were incubated in 37 °C for 30 min. For the last 3 min, we added 1 μM lysotracker (Beyotime, C1046) inside the wells. After staining, the cells were washed two times with fresh Dulbecco's Modified Eagle's Medium (DMEM) and then observed by imaging. After searching for some target cells in the region of interest, we added 100 μl PKH67-labeled exosomes inside the light-sheet chamber (the buffer volume was around 4 ml), and then observed using imaging for 60 min (the imaging speed was 1 min per frame).

### Enzyme-linked immunosorbent assay (ELISA) analysis

Conditioned culture medium was harvested from BV2 cell cultures after exposure with control or exosomes for 24 h. TNF-α (Bio-Awamp, MU30030) and IL-6 ELISAs (Bio-Awamp, MU30044) were performed according to manufacturer’s instructions. Total α-syn (Bio-Awamp, HM10821) in exosome was quantified by human-specific ELISA kit, according to manufacturer’s instructions.

### Thioflavin T assay

One-hundred microliters of each exosomes sample were mixed with Thioflavin T to obtain a final concentration of 10 mM and then were incubated in a 96-well plate at room temperate for 10 min. The fluorescence was measured on a Multi-Mode Microplate Reader with an exicitation at 445 nm and an emission at 480 nm.

### Western blot analysis

Samples were digested with RIPA lysis buffer containing peotease inhibitor cocktail and phosphatase inhibitor cocktail. For Western blot, samples were mixed with sodium dodecyl sulfate (SDS) loading buffer, and separated on 10% or 12% SDS-polyacrylamide gel electrophoresis (PAGE). Proteins were transferred onto polyvinylidene difluoride membranes. Membranes were blocked in 5% (w/v) powdered skimmed milk in Tris-buffered saline/0.5‰ Tween 20 (TBS-T) for 1 h. Incubation with primary antibodies was performed overnight at 4 °C. On the next day, after three 8 min washing steps in TBS-T, blots were incubated with secondary horseradish peroxidase-coupled antibodies for 1 h at room temperature. After three 8 min washing steps in TBS-T, western blots were revealed by chemiluminescence. The protein levels were quantified by densitometry using ImageJ v1.47 software (National Institutes of Health, USA).

The following primary antibodies were utilized: rabbit antibody to IBA1 (1:1000, 10904-1-AP, Proteintech), mouse antibody to tsg 101 (1:500, sc-7964, Santa cruz biotechnology), mouse antibody to CD63 (1:1000, ab59479, Abcam), rabbit antibody to Calnexin (1:1000, 10427-2-AP, Proteintech), rabbit antibody to α-syn (1:1000, ab138501, ab52168, Abcam), rabbit antibody to Oligomer (1:1000, AHB0052, ThermoFisher scientific), rabbit antibody to TH (1:1000, 25859-1-AP, Proteintech), rabbit antibody to LC3 (1:1000, 14600-1-AP, Proteintech), rabbit antibody to SQSTM1/P62 (1:1000, ab91526, Abcam), rabbit antibody to Beclin1 (1:1000, 11306-1-AP, Proteintech), rabbit antibody to phosphor-AKT (1:800, AP0655, Abclonal), rabbit antibody to AKT (1:800, A11030, Abclonal), rabbit antibody to phosphor-mTOR (1:400, BM4840, Boster), mouse antibody to α-syn (1:1000, synuclein Ab-2, Thermo Scientific), and rabbit antibody to p-syn (1:500, GTX50222, Genetex).

### Cell culture

BV2 murine microglial cell line was cultured in DMEM/hign-glucose medium with 10% fetal bovine serum. HM was cultured in DMEM/high-glucose medium with 10% fetal bovine serum. SH-SY5Y human neuroblastoma cell line was cultured in DMEM/F12 medium with 10% fetal bovine serum. The cells were maintained at 37 °C in a humidified incubator with an atmosphere of 5% CO_2_.

### Stereotaxic injections

Unilateral intrastriatal injection of exosomes was performed on anesthetized mice using stereotaxic apparatus at the following coordinates (relative to Bregma): AP, −0.4 mm posterior to bergma; ML, 1.8 mm lateral to the midline; DV, −3.5 mm vertical from the dura. One or two weeks postinjection mice were anesthetized and transcardial perfusion with 0.9% saline followed by 4% paraformaldehyde in PBS. Brains were removed and postfixed overnight in 4% paraformaldehyde in PBS for cryostat sectioning.

### Immunofluorescence

The immunofluorescence staining was performed on 10-μm-thick serial frozen sections of brain tissue. Sections were treated with 0.5% H_2_O_2_ in methanol to inactivate peroxidase. After blocked with 10% donkey serum, brain sections were incubated with primary antibodies at 4 °C overnight, then processed with secondary antibody conjugated to Alexa Fluor 488 or 594. Nuclei were stained with 4′, 6-diamidino-2-phenylindole (DAPI). Sections were imaged with Nikon A1-Si confocal microscope. Cells were seeded onto coverslips in 24-well plates and treated with exosomes derived from plasma for 24 h. Cells were then fixed, permeabilized, blocked, and then stained as previously described.

For immunofluorescence, primary antibodies were rabbit antibody to IBA1 (1:200, 10904-1-AP, Proteintech; 1:500, NO.019-19741, Wako), rabbit antibody to GFAP (1:200, 16825-1-AP, Proteintech), rabbit antibody to MAP2 (1:200, 17490-1-AP, Proteintech), mouse antibody to α-syn (1:100, sc12767, Santa cruz biotechnology), rabbit antibody to p-syn (1:400, 23706S, Cell Signaling Technology), mouse antibody to LAMP1 (1:200, sc20011, Santa cruz biotechnology), sheep antibody to TH (1:500, ab113, Abcam), rabbit antibody to SQSTM1/P62 (1:1000, ab91526, Abcam), and rabbit antibody to NeuN (1:300, ab177487, Abcam).

### Statistical analysis

All values were expressed as mean ± SEM of at least three independent differentiations. All data were analyzed by unpaired two-tailed *t* test with Welch correction for heteroscedasticity. All statistics were calculated using GraphPad Prism software (San Diego, CA, USA) and a value of *p* < 0.05 was considered statistically significant.

## Results

### Plasma exosomal α-syn in individuals with or without PD

**Fig. 1:**
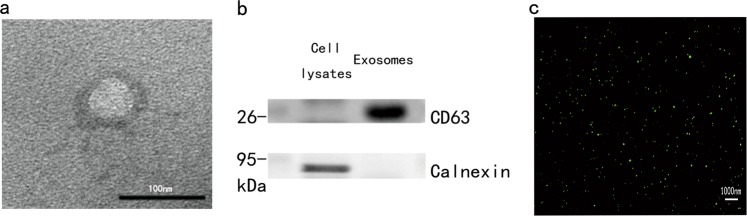
Characterization of exosomes isolated from human plasma. **a** Representative transmission electron microscopy (TEM) observation of exosomes isolated from human plasma, Scale bar = 100 nm. **b** Western blot shows the presence of exosomal marker CD63 and the absence of negative marker Calnexin in plasma-derived exosomes. **c** Super-resolution imaging was performed to visualize the density of labeled-exosomes (green) in the exosome preparations

**Fig. 2:**
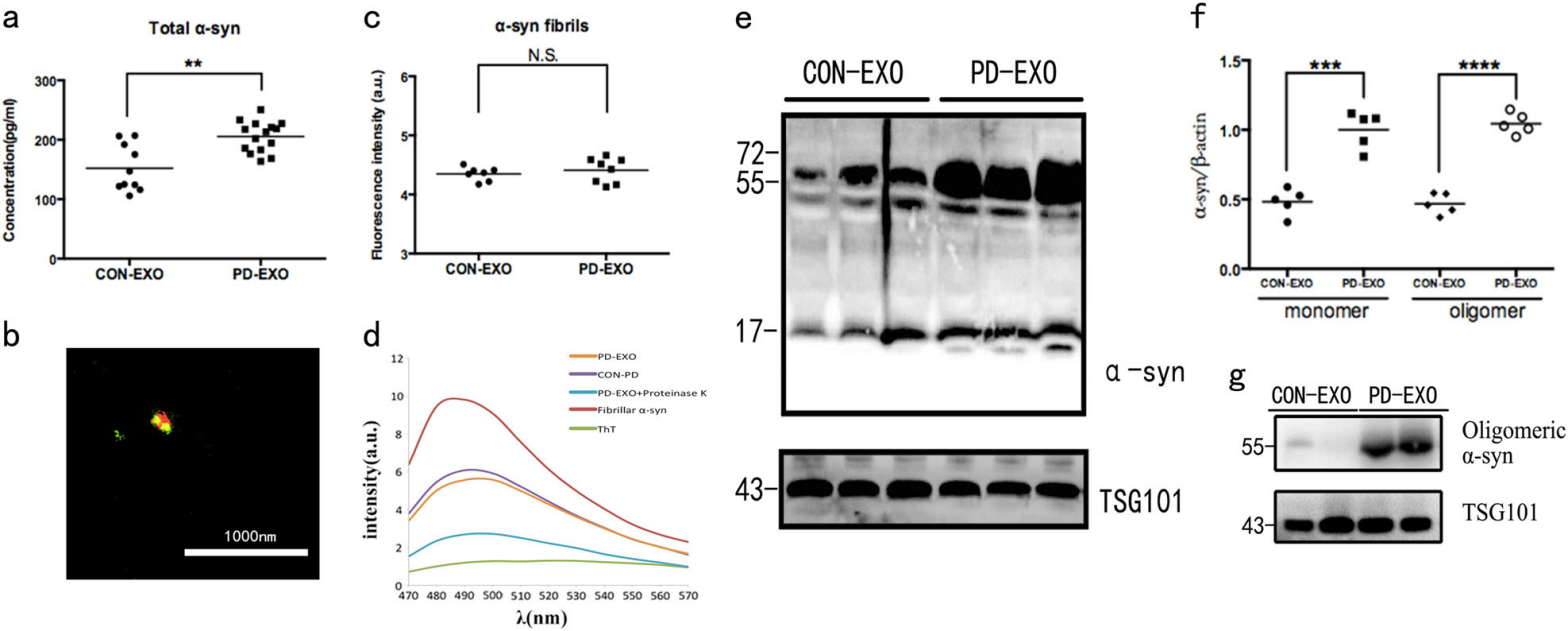
Levels of α-syn in plasma-derived exosomes of patients with PD (PD-EXO) and normal controls (CON-EXO). **a** Concentrations of exosomal total α-syn in plasma determined by ELISA (PD patients, *n* = 15; healthy controls, *n* = 10; **p = 0.02). **b** Representative image of double immunolabeling analysis for α-syn (red) with either lipophilic dye DiD (green) in exosomes. DiD-labeled exosomes showed immune-reactivity against α-syn antibody. Images were captured on a super-resolution microscopy. Scale bar = 1000 nm. **c** Thioflavine-T (ThT)-assay shows no difference of fibrillar α-syn in both groups (PD patients, *n* = 8; healthy controls, *n* = 7; *p* = 0.4729). **d** Representative ThT fluorescence emission spectra of the exosomes or fibrillar α-syn samples. **e** Immunoblots show the levels of oligomeric and monomeric exosomal α-syn expression (*n* = 5; monomers: ***p = 0.0002; oligomers: ****p < 0.0001). Bolts were probed for TSG101 as a loading control. **f** The quantitative data of monomeric and oligomeric exosomal α-syn. **g** Representative immunoblots show oligomeric α-syn expression in the exosomes derived from plasma. Oligomeric human α-syn was probed using the Oligomer A11 Antibody, which recognizes amino acid sequence-independent oligomers of α-syn, but not monomers and mature fibers. TSG101 is shown as a loading control

The exosome-rich fraction was purified from the plasma of PD patients and healthy controls using differential ultracentrifugation. Twenty sporadic mild-late stage PD patients (14 male, 6 female, mean age 70.0 years, mean disease duration 11.5 years) were registered (Table 1). We used transmission EM to observe the size (40–100 nm diameters) and the cup-shaped morphology of exosomes (Fig. 1a). As expected, CD63 and TSG101, two commonly used marker proteins for exosomes, were highly enriched in the exosomes preparations compared to the cell lysates, indicating the high purity of exosomes (Fig. 1b, Fig. 2c, d). In addition, calnexin, an endoplasmic reticulum protein, was only detectable in cell lysates, demonstrating an absence of cellular components or other vesicles in the exosomes preparations (Fig. 1b). Moreover, super-resolution microscopy was used to enable visualization of the exosomes density in solution (Fig. 1c).

**Table 1:** Clinical characteristics of patients

Subject no.	Age (years)	Gender (M/F)	Age at onset	UPDRS_III	H&Y
1	63	M	45	28	3
2	63	F	58	54	3
3	69	M	58	32	4
4	64	M	51	39	3
5	80	M	72	26	3
6	70	M	49	59	3
7	68	F	58	52	3
8	61	M	50	42	3
9	81	F	70	55	3
10	67	M	56	56	3
11	70	M	60	28	3
12	72	F	62	50	3
13	69	M	62	46	3
14	68	M	58	42	3
15	71	F	60	51	3
16	68	M	58	42	3
17	69	M	58	32	4
18	82	F	72	38	2
19	80	M	64	36	3
20	65	M	49	40	3

The concentrations of total α-syn in plasma-derived exosomes were determined by ELISA and were found to be significantly higher in PD exosomes compared to the controls (Fig. 2a). Interestingly, the positive signal obtained using antibody α-syn in super-resolution imaging, suggested that α-syn is partly exposed on the surface of plasma exosomes derived from PD patients (PD-EXO) (Fig. 2b). Next, we used the thioflavin T (ThT)-assay to analyze the fibrillar strutures of exosomal α-syn; however, there was no significant difference observed in plasma exosomal fibrillar α-syn concentrations in PD vs. controls (Fig. 2c). To confirm that the detected signal represents real α-syn fibrils, we used commercial preformed α-syn fibrils as a positive control (Fig. 2d). The commercial fibrils showed the same spectrum characterize of the exosomes, indicating that the detected signal reflect α-syn fibrils in the exosomes. To further confirm the signal was α-syn fibrils, we treated the exosomes with protease K to digest the fibrils, and tested the ThT signal again. As expected, the proteased K-digested samples showed much lower signaling compared to control, further supporting the specificity of the ThT signal. Furthermore, exosomal proteins were fractionated to determine the level of oligomeric and monomeric α-syn using SDS/PAGE (Fig. 2e). Plasma-derived exosomes from PD patients contained more amounts of α-syn oligomers and monomers compared to control exosomes (Fig. 2f). To further reconfirm the presence of oligomers, we compared the immunoreactivity of exosomal α-syn by an oligomer-specific A11 antibody (Fig. 2g). Western blot analysis indicated a strong reaction.

### Intrastriatally infused exogenous exosomes are taken up by microglia and transported to substantia nigra and cortex

**Fig. 3:**
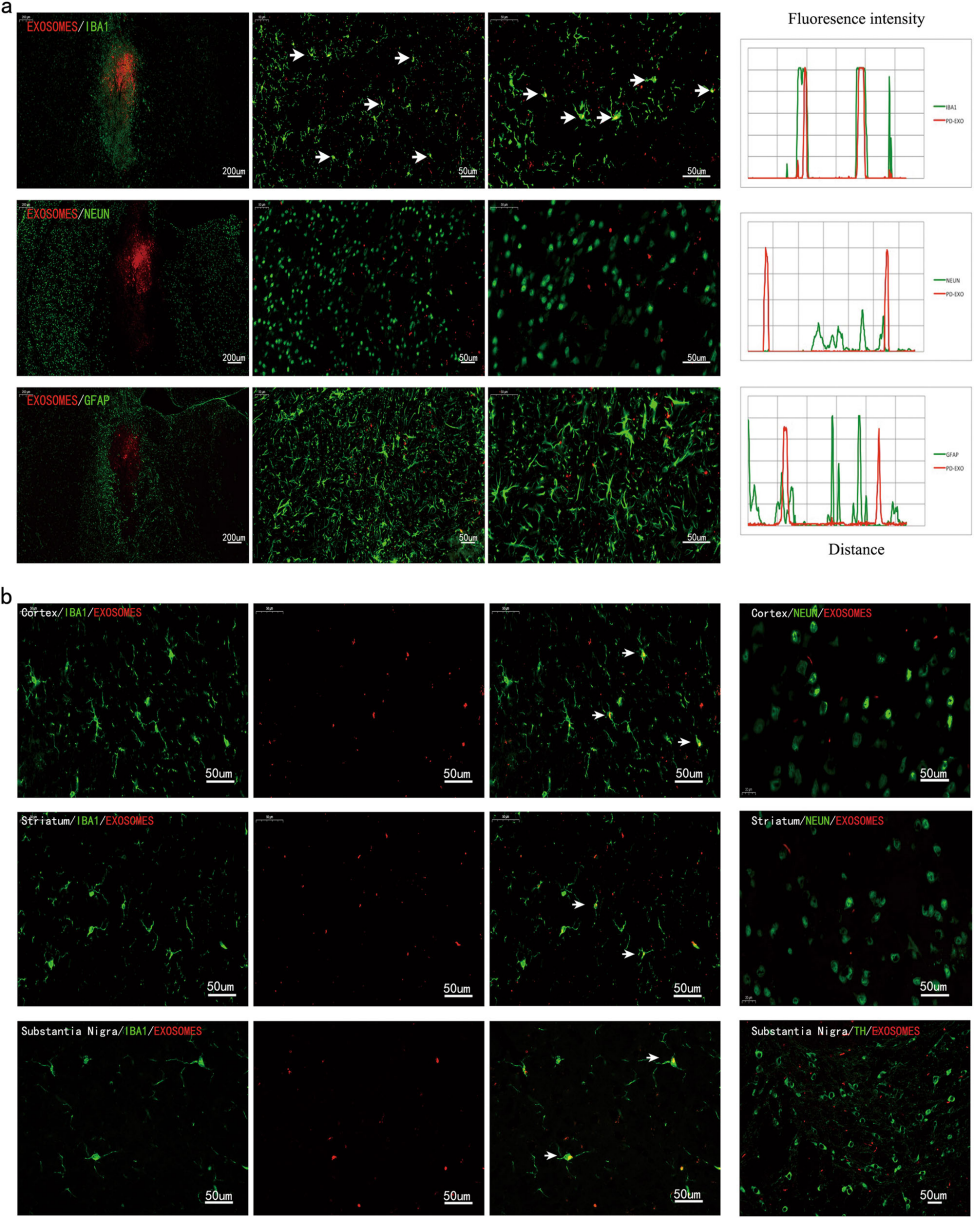
Plasma exosomes derived from PD patients preferentially target microglia in vivo, rather than neuron or astrocyte. The brain tissues were harvested from mice 1 week after unilateral intrastriatal injection of PKH26-labeled exosomes. **a** Representative immunofluorescent images showed colocalization of exosomes with the marker of microglia IBA1 in the wild-type mouse brain. Labeled-exosomes were taken up by microglia neighboring the injection site. Neuron labeled with the neuronal marker NEUN and astrocyte with GFAP show phagocytosis deficit of exosomes surrounding the injection site. Right graphs: representative fluorescence intensity profiles of neural specific markers (green) and PKH26-labeled exosomes (red) in the partial regions of the high-power magnification image. Confocal analysis showed partial colocalization of PKH26-labeled exosomes and IBA1, whereas no colocalization was detected with NEUN and GFAP. **b** Intrastriatally administered PKH26-labeled exosomes diffuse through the mouse brain, localizing to the cortex, striatum, and substantia nigra. Double immunofluorescence staining was performed with IBA1 (green) antibody and exosomes (red). White arrowheads indicate the phagocytosis of labeled-exosomes by microglia. No labeled-exosome was co-localized with NEUN-labeled neurons or TH-labeled neurons. TH, tyrosine hydroxylase, a marker of dopamine neurons (green)

In order to visualize the spread and clearance of plasma exosomes derived from PD patients, we labeled the exosomes with a lipophilic fluorescent linker PKH26, and subsequently infused them stereotaxically into the unilateral striatum of 8-month-old mice. After 1 week of exposure, brain sections were double-stained with the neural marker antibodies of brain cells and DAPI. We observed large numbers of microglial cells and astrocytes that accumulated at the injection site of striatum (Fig. 3a). Entrance of exosomes into the microglial cells was further confirmed by immunofluorescent staining showing colocalization of red fluorescent exosomes and IBA1 staining. Confocal imaging showed that the microglial cells, but not neurons and astrocytes, were able to take up large amounts of exosomes. Moreover, we studied the brain distribution of exogenous exosomes in vivo. Microglial cells in the bilateral striatum, substantia nigra, and cortex all showed cellular internalization of PKH26-labeled exosomes (Fig. 3b). Despite the unilateral inoculation, PKH26-labeled exosomes deposits were widely distributed bilaterally. This preferential internalization suggested that microglia have a certain degree of affinity for the plasma exosomes derived from PD patients.

### Plasma exosomes derived from PD patients efficiently enter into microglial cells and induce microglial activation in vitro and in vivo

**Fig. 4:**
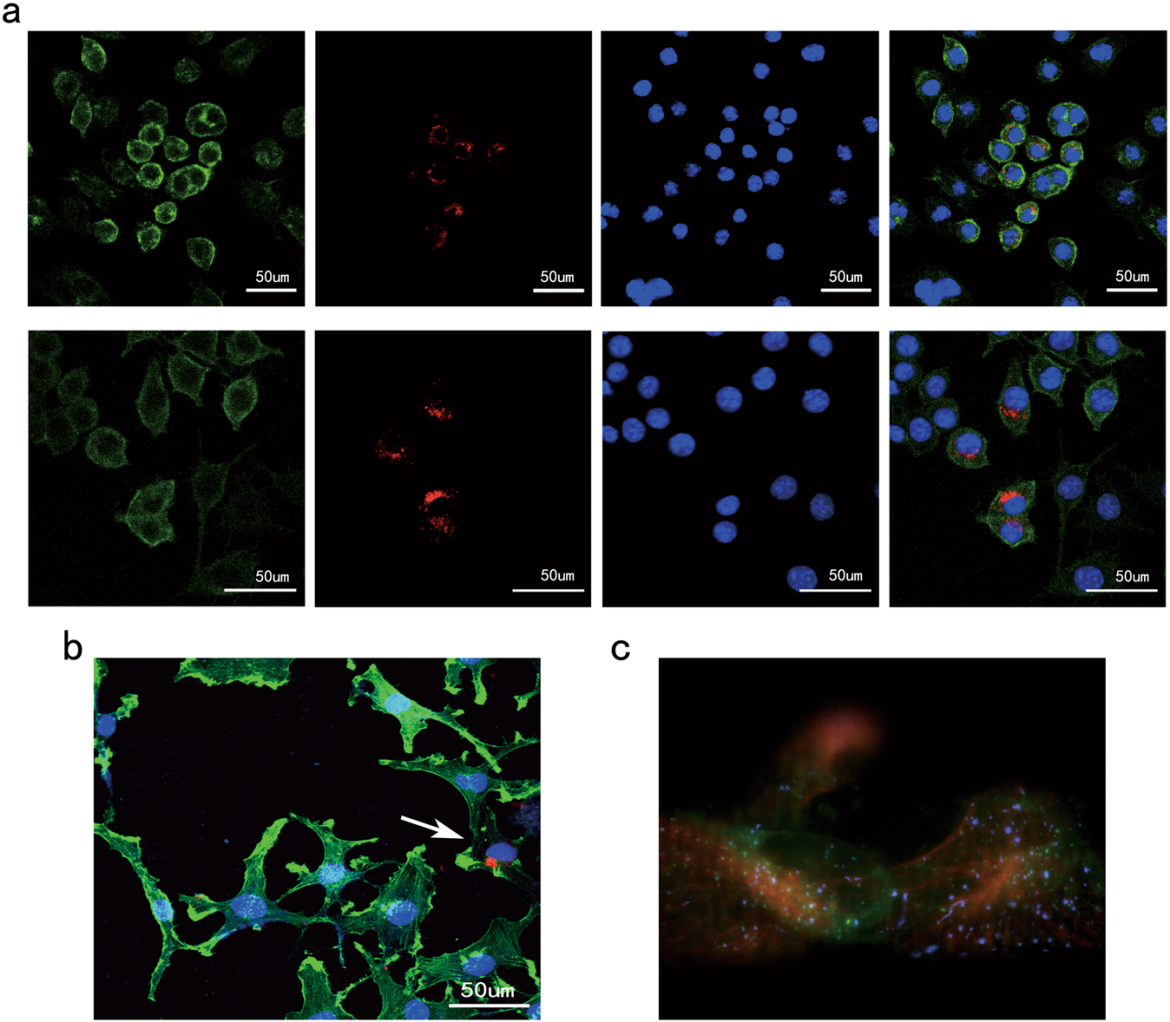
Uptake of PKH-labeled plasma exosomes derived from PD patients by microglial cells in vitro. **a** BV2 cells were co-cultured with PKH26-labeled exosomes for 12 h at 37 °C. For microscopic analysis, BV2 cells were visualized using the green fluorescent lipophilic dye DIO. **b** HM cells were co-cultured with PKH26-labeled exosomes for 12 h before fixing and staining with phalloidin (green) and DAPI (blue). **c** The dynamic uptake of PKH67-labeled exosomes in HM cells was detected by super-resolution live cell imaging. Blue channel: the plasma exosomes derived from PD patients; green channel: lysosome; red channel: tubulin (see the video section)

To determine whether plasma exosomes derived from PD patients enter the microglial cell line BV2, PKH26-labeled exosomes were added to the culture medium. We then fixed BV2 cells at 12 h after exposure to exosomes, stained them with membrane dye, and then analyzed by confocal microscopy (Fig. 4a). We firstly provided in vitro supporting evidence by observing the internalization of labeled exosomes by BV2 cells. Efficient uptake of exosomes was also detected when HM was used (Fig. 4b). Furthermore, by using light sheet-based fluorescent microscopy, we described a method for the three-dimensional live imaging of the dynamics pertaining to exosome internalization and trafficking (Fig. 4c, play video with Image J in Supplementary Data). Light-sheet microscopy enables deep-cell super-resolution imaging of exosomes in live HM. Intriguingly, HM cells, cultured in vitro, rapidly internalize labeled exosomes that are added to the medium. A more detailed analysis of light-sheet live cell imaging revealed accumulations of exogenous exosomes inside the cytoplasm, within close proximity of the lysosomes.

**Fig. 5:**
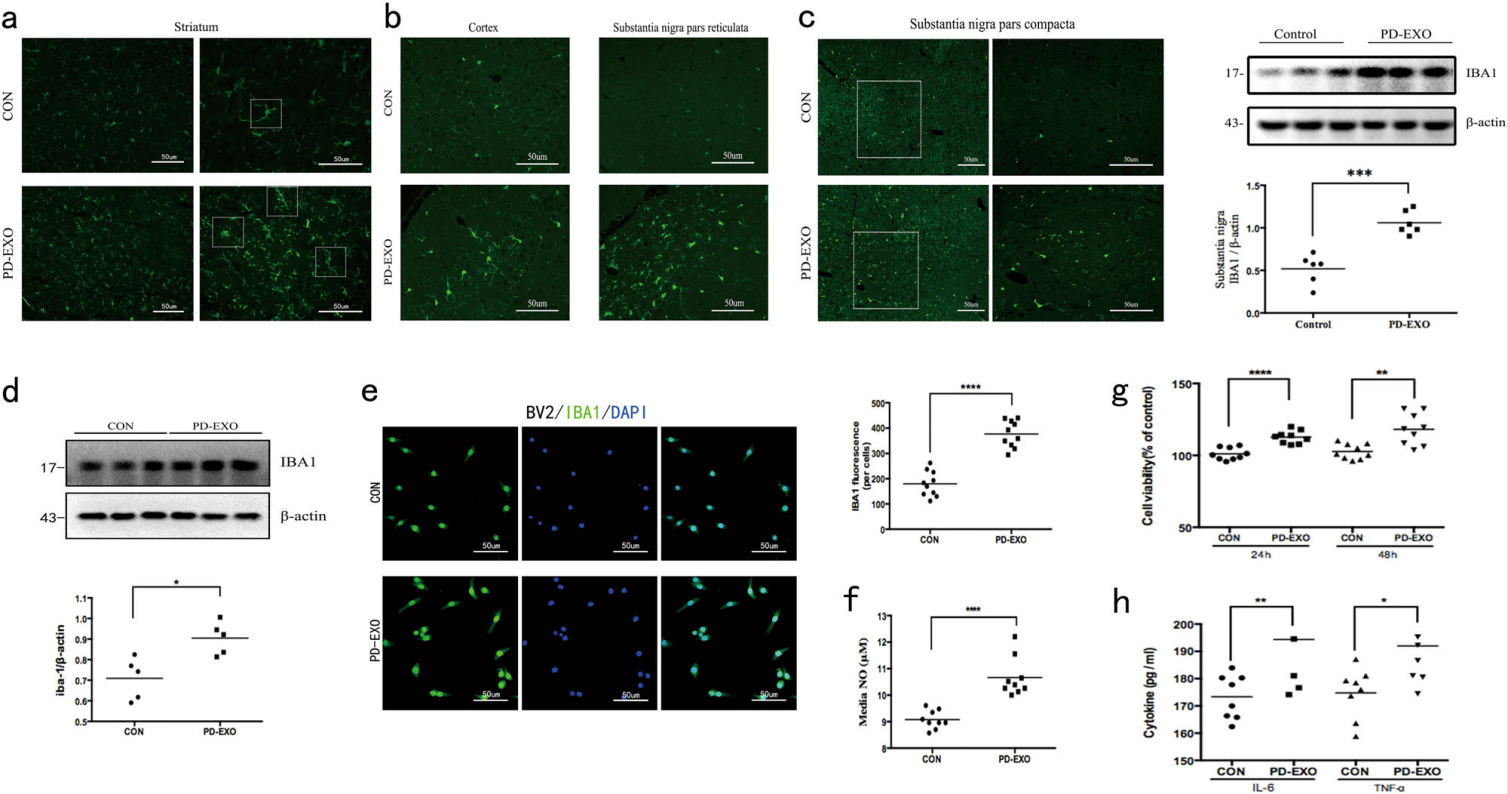
Plasma exosomes derived from PD patients activate microglia in vivo and in vitro. **a** Comparison of IBA1 immunofluorescence staining of reactive microglia. Representative images of the ipsilateral striatum of Wild-type mice stereotaxically injected with PBS or PD-EXO and sacrified 2 weeks later. Intrastriatally injected exosomes caused morphological change and proliferative activity of microglia at the injection site. Squares show detailed morphology of the microglia phenotype stained with IBA1. **b** Representative immunofluorescent staining of IBA1 in the cortex and substantia nigra pars reticulate of wild-type mice stereotaxically injected with PBS or PD-EXO. **c** Representative images comparing IBA1 expression of the substantia nigra pars compacta (SNpc) after intrastriatal injection of either PBS or PD-EXO (*n* = 3, ***p = 0.001). **d**–**h** The BV2 cells were stimulated with exosomes for 24 h, and the culture supernatants were collected. **d** Western blot analysis (*n* = 5, *p = 0.01) was performed to analyze the alterations in the protein expression of IBA1. **e** Microglia activation was evaluated through determination of IBA1 expression using immunofluorescence assays (*n* = 10, ****p < 0.0001). **f** The supernatants were subjected to detect nitric oxide by Griess reagent system (*n* = 9, ****p < 0.0001). **g** Plasma exosomes derived from PD patients induced the proliferation of BV2 cells, which was measured using CCK8 assay (*n* = 9, 24 h: ****p < 0.0001; 48 h: **p = 0.0027). **h** The supernatants were subjected to ELISA assays for determining production of tumor necrosis factor-α and interleukin-6 (*n* = 8), TNF-α (*p = 0.0168) and IL-6 (**p = 0.0059) secretion (by ELISA assays)

α-syn is a classical molecular contributor in PD and plays an important role in the occurrence of microglial activation^29,30,42–44^. To investigate the effect of exosomes that contained α-syn on microglial activation, we quantified the degree of microglial reactivity. Firstly, microglial activation was assessed at the injection site through the determination of IBA1 expression (Fig. 5a). The results of immunofluorescence identified that, compared to PBS-treated mice, exosome-exposed ones showed a markedly increased IBA expression, and the individual microglia showed a greater activated phenotype. In addition, increased expression of IBA1 has been identified in the cortex and substantia nigra (Fig. 5b, c). In case of PD-EXO, our results indicate that microglia could switch to an activated and phagocytic state.

To further determine the role of exosome in microglial activation, we examined its influence on the IBA1 expression, cell viability, NO formation, and inflammatory cytokine secretion in BV2 microglial cells. Immunofluorescent staining and western blotting of IBA1 demonstrated that the levels of IBA1 significantly increased at 24 h following exosomal stimulation (Fig. 5d, e). Moreover, plasma exosomes derived from PD patients caused significant elevation of NO production, proliferation, and cytokine secretion (Fig. 5f–h). Consequently, our results indicated a major influence of plasma exosomes derived from PD patients in the regulation of microglial activation and proinflammatory mediator production.

### Plasma exosomes derived from PD patients are involved in the cell-to-cell transmision of α-syn in vivo

**Fig. 6:**
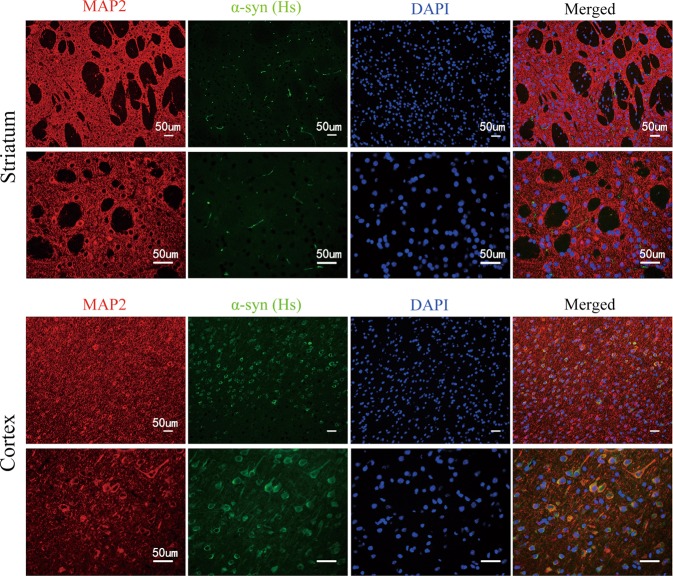
Transfer of plasma exosomal α-syn from the striatum to the ipsilateral cortex in mice. The brain tissues were harvested from mice 2 weeks after unilateral intrastriatal injection of exosomes derived from PD patients. **a** Representative images of double immunolabeling analysis for α-syn (human-pecific antibody) with either neuronal marker MAP2 at the injection site, 2 weeks postinjection. **b** Representative images of double immunolabeling analysis for α-syn (human-specific antibody) with either neuronal marker MAP2 in the cortex, 2 weeks after injection. Human α-syn was partly co-localized with the neuronal marker MAP2

Recent studies have revealed that exogenous pathogenic α-syn have prion-like properties, which are characterized by cell-to-cell transmission and propagation of misfolded proteins^45–48^. Based on these findings, we hypothesized that intrastriatal inoculation of plasma exosome-associated α-syn, derived from PD patients, could been transferred to recipient cells in vivo. In order to directly monitor plasma exosome-associated α-syn, we assessed the presence of α-syn in the brain section by immunofluorescence assay using an antibody that specifically recognizes human α-syn. As expected, human-specific α-syn was readily detected at the injection site (Fig. 6a). However, MAP2 positive neurons were rarely co-localized with human α-syn in the striatum. Strikingly, exogenous human α-syn were not restricted to the injection sites but progressed over time to the neighboring cortex (Fig. 6b). Positive human α-syn signal was detected, partly co-localizing with the neuronal marker MAP2 in the cortex.

**Fig. 7:**
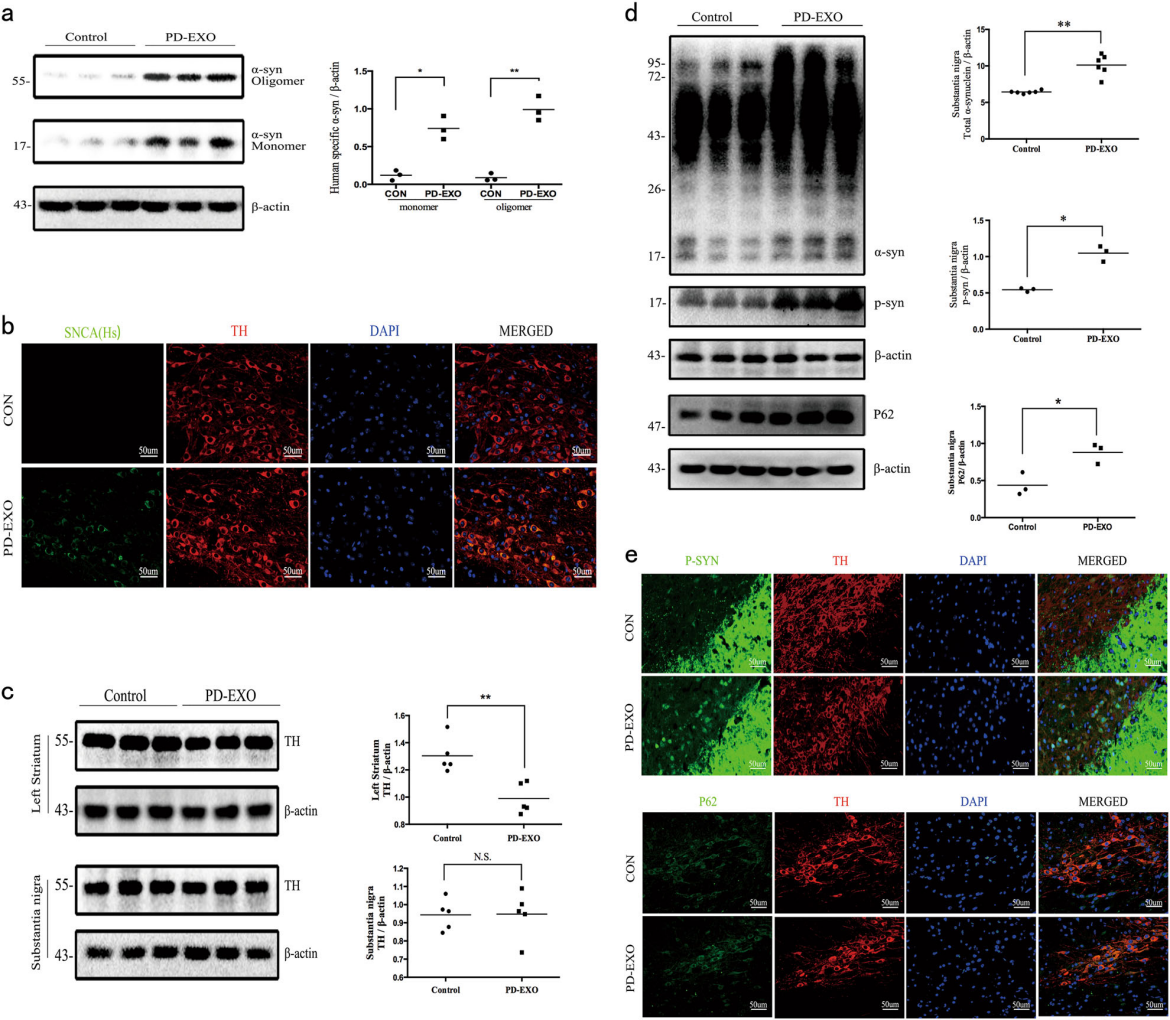
Plasma exosomal α-syn transimission initiates the aggregation of endogenous and exogenous α-syn in the SNpc of mice. **a**, **b** Human-specific α-syn immunobloting and immunostaining in SNpc of wild-type mice sacrificed at 2 weeks after unilateral injection of human plasma exosomal α-syn into the striatum. PBS was inoculated to the control animal. **a** Staining with human-specific α-syn antibody detects exogenous injected α-syn (*n* = 3, monomer: *p = 0.0105, oligomer: **p = 0.0065). **b** Double immunostaining for human-specific α-syn (green) and TH (red) demonstrated colocalization of human-specific α-syn and TH in the nigral neurons. **c** TH expression in striatum and SNpc was evaluated by western blot (*n* = 5, striatum: **p = 0.0034, SNpc: *p* = 0.9554). **d** Seeding of endogenous α-syn in SNpc by exogenous human exosomal α-syn. Immunoblots of brain homogenates extracted from the treated SNpc were stained with α-syn antibody that recognized both human and mice α-syn (*n* = 6, **p = 0.0013). The expression of phosphorylated-α-syn (p-syn) (*n* = 3, *p = 0.0113) and P62/SQSTM1 (*n* = 3, *p = 0.0211) in the SNpc were determined by western blot and immunofluorescence (**e**)

Moreover, human α-syn is localized to dopaminergic neurons of the SNpc (Fig. 7a, b). As the plasma exosomal α-syn can spread over considerable distances to many brain regions, we investigated whether exogenous α-syn can induce α-syn pathology in vivo. In the current study, we showed that the endogenous α-syn is extensively oligomerized after the internalization of human α-syn (Fig. 7d). Since the aggregation of α-syn is accompanied by the development of α-syn hyperphosphorylation, we investigated whether α-syn was phosphorylated in dopaminergic neurons. Phosphorylated α-syn is abundantly detected in dopaminergic neurons of the SNpc as expected (Fig. 7d, e). Accumulation of P62/SQSTM1 was also apparent in dopaminergic neurons of the SNpc (Fig. 7d, e). Notably, intrastriatal inoculation of human exosomal α-syn significantly reduced the expression of tyrosine hydroxylase of striatum dopaminergic neurons but not of SN dopaminergic neurons (Fig. 7c). This phenomenon may be related to the pattern of short-period administration. These results strongly suggested that human α-syn propagates throughout the brain from the injection site. The extensive colocalization between exogenous human α-syn and neurons further supports the ability of specific exosome-associated α-syn conformers that are capable of microglia-to-neuron transmission.

### Plasma exosomes derived from PD patients inhibit autophagy and accelerate α-syn accumulation in BV2 cells

**Fig. 8:**
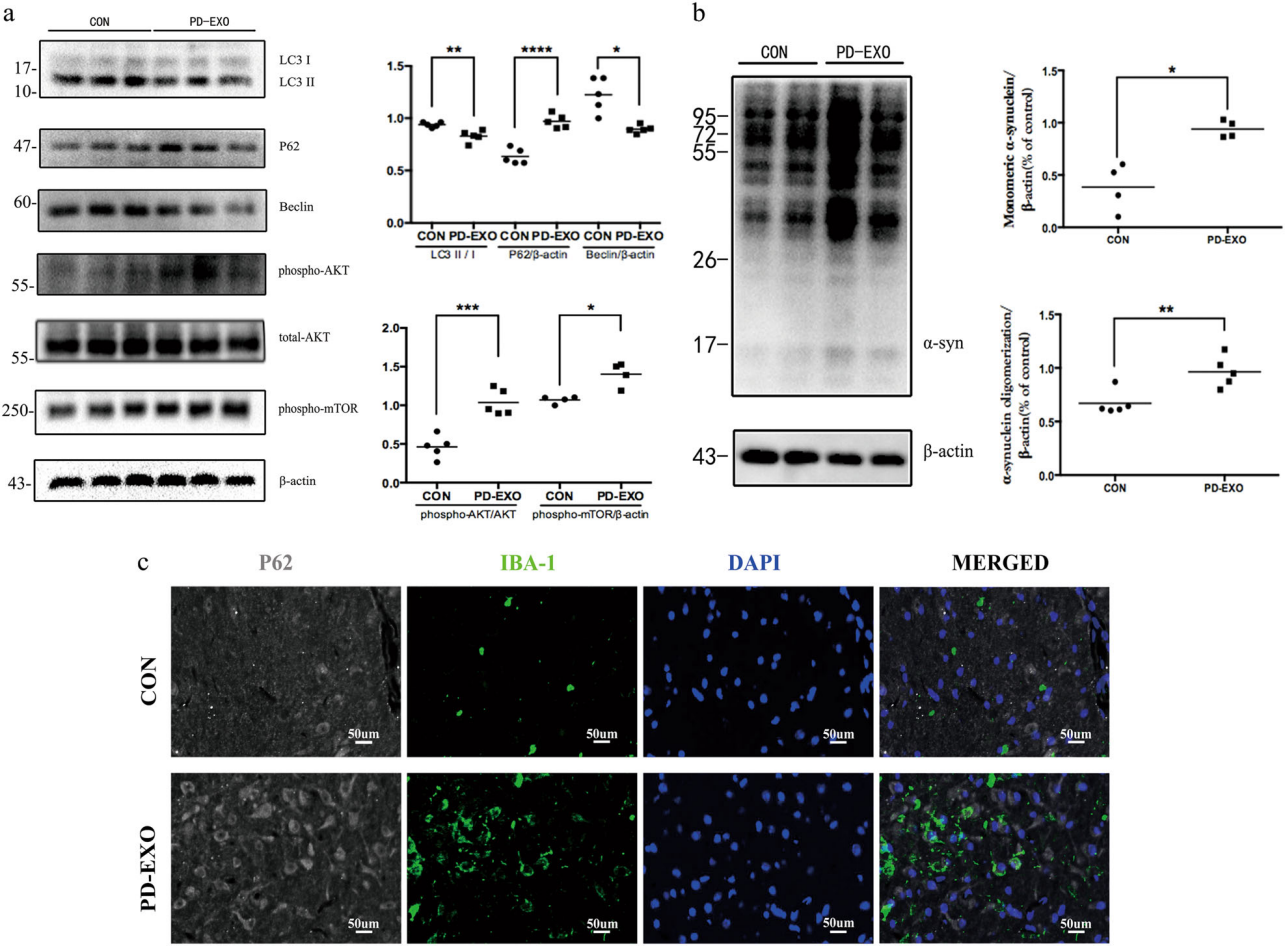
Plasma exosomes derived from PD patients inhibit autophagy and induce accumulation of α-syn in BV2 cells. Whole-cell lysates were analyzed by western blot. **a** Protein levels of LC3 (*n* = 5, **p = 0.0082), P62/SQSTM1 (*n* = 5, ****p < 0.0001), Beclin (*n* = 5, *p = 0.0101), phospho-AKT (*n* = 5, ***p = 0.0004), total-AKT, phospho-mTOR (*n* = 4, *p = 0.0183), and β-actin were shown in indicated cells. **b** Whole-cell lysates were immunoblotted against α-syn (oligomeric) (*n* = 5, **p = 0.0078); monomeric (*n* = 4, *p = 0.0113) and β-actin for control. **c** The triple-stained image of P62/SQSTM1 (gray) with IBA1 (green) and DAPI (blue) in the SNpc

Autophagy refers to a conserved degradation system that clears malformed or aggregated proteins within cells^49^. Much evidence demonstrates that insoluble or aggregated α-syn is targeted in the autophagy-lysosome pathway^50–53^. To investigate the role of autophagy in exosome-associated inflammatory responses of BV2 cells, western blotting was used to assess the change in expression levels of the LC3 II, P62, and Beclin1 proteins (Fig. 8a). Results showed that the protein levels of LC3 II and Beclin1 decreased in exosomes-treated BV2 cells, compared to an increased level of P62 proteins, indicating the inhibition of autophagy. We then assessed autophagic activity using P62 immmunostaining in the SNpc of mice (Fig. 8c). Moderate P62 expression in the microglial cells of SNpc was observed only in the PD-EXO injection mice.

To address whether the Akt-mTOR signaling pathway is involved in exosome-inhibited autophagy, we investigated the expression of key proteins in this pathway by western blot (Fig. 8a). Results revealed a progressive increase in the expression of phosphorylated mTOR in the exosome group compared to the control. To further determine the upstream activation of mTOR, we detected Akt phosphorylation, and consistent result was found in the expression of phosphorylated Akt. Therefore, plasma exosomes derived from PD patients promotes the activation of the Akt-mTOR signaling and suppresses autophagy in BV2 cells.

As autophagy plays a crucial role in α-syn degradation, we speculate that the dysregulation of exosome-associated autophagy contributes to the accumulation of α-syn in BV2 cells. In this study, we observed a significant elevation to the level of oligomeric and monomeric α-syn protein in exosome-treated cells as compared to nontreated BV2 cells (Fig. 8b). As expected, plasma exosomes derived from PD patients lead to α-syn accumulation in BV2 cells.

### Exosomes released under PD-EXO treatment contain higher α-syn cargo in vitro

**Fig. 9:**
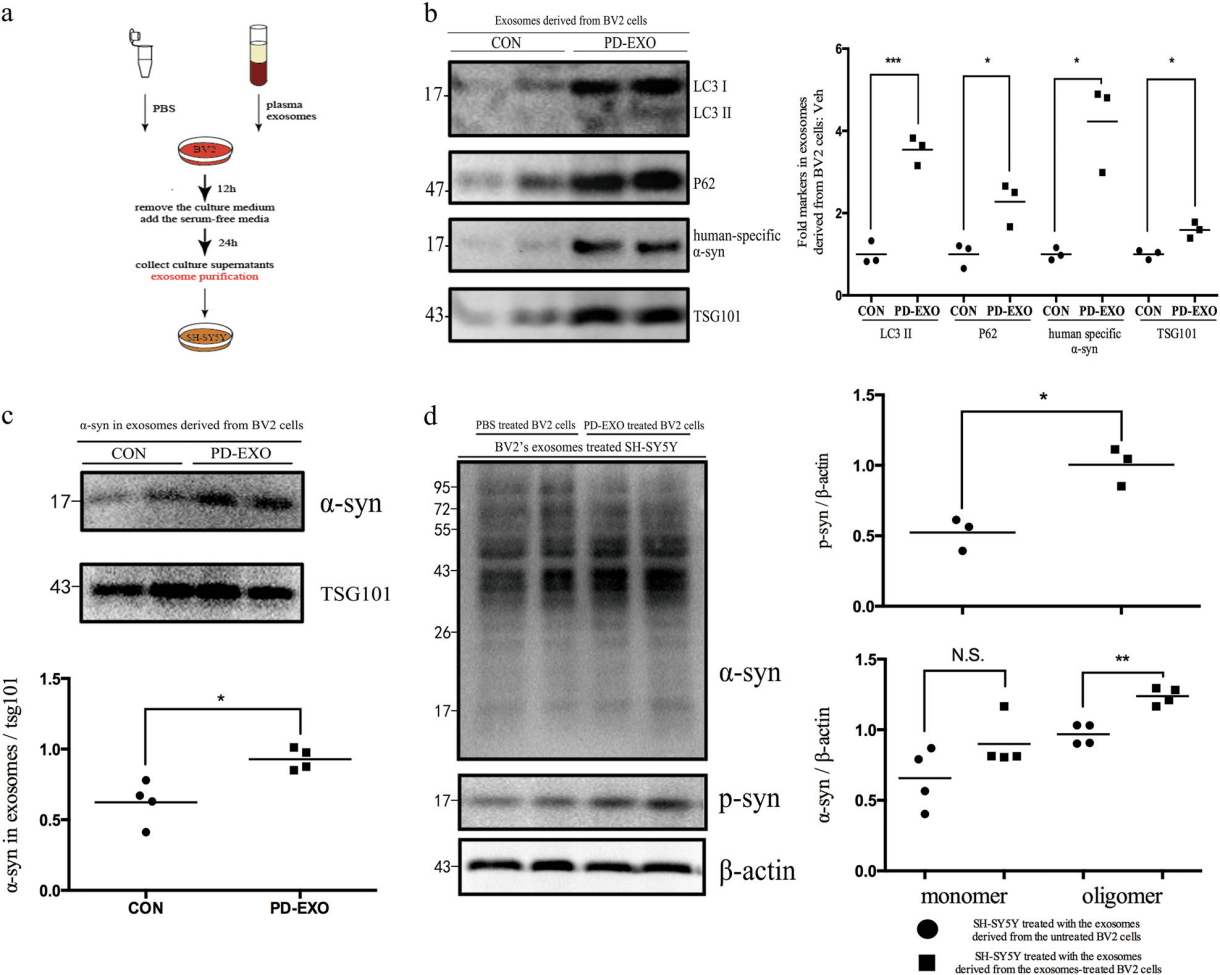
Plasma exosomes derived from PD patients modulate α-syn release in exosomes derived from BV2 cells, thereby affecting intercellular communication. BV2 cells were treated with PBS or plasma exosomes derived from PD patients. **a** Schematic experimental settings for exosomes derived from BV2 cells harvest. **b** Increased autophagosome markers in exosomes derived from BV2 cells under PD-EXO intervention. Two groups of exosomes suspensions were extracted from the same volume of BV2 cells culture supernatant. Equal exosomes volumes were analyzed on the same blot. Immunoblots for LC3 (*n* = 3, ***p = 0.0007) and P62 (*n* = 3, *p = 0.0334) and TSG101 (*n* = 3, *p = 0.0167) in exosomes derived from the culture media of BV2 cells treated with PBS and PD-EXO as indicated. Immunoblots of exosomes derived from BV2 cells culture media were stained with an α-syn antibody specific for human α-syn (**b**) (*n* = 3, *p = 0.0329) and an α-syn antibody that recognized both human and mice α-syn (**c**). **c** Western blot analysis of exosomes isolated from BV2 cells show that α-syn was detected in both fractions and increased by PD-EXO (*n* = 4, *p = 0.0203). **d** Western blot of α-syn expression of SH-SY5Y cells exposed to extracellular exosomes derived from BV2 cells. Expression of aggregated form of α-syn increased (*n* = 4, **p = 0.0015). Similar result was detected in phosphorylated α-syn expression (*n* = 3, *p = 0.0100), but not monomeric α-syn (*n* = 4, *p* = 0.1329)

Emerging evidences suggest that exosomal secretion and autophagy–lysosomal pathways are coordinated to remove α-syn protein aggregates^7,10,11,54,55^. Moreover, it was reported that autophagy–lysosomal pathway dysfunction could promote exosome-mediated α-syn release. To test this view, exosomes of treated BV2 cells were isolated from the conditioned supernatant by ultracentrifugation (Fig. 9a). Human-specific α-syn has been identified in exosomal fraction derived from cell culture supernatant in PD-EXO treated BV2 cells (Fig. 9b). Thus, exosomes of PD-EXO treated BV2 cells may serve as seeds for subsequent cell-to-cell transmission.

BV2 cell culture supernatant of control group was separated in identical volumes and exosomes were collected. Within exosomes from two groups of BV2 conditioned medium, we observed that approximately 1.6-fold the exosomal marker TSG101 upregulation was quantified under PD-EXO treatment, suggesting that exosome abundance was enhanced. Moreover, the addition of PD-EXO led to an increase in the levels of exosome-associated monomeric α-syn (Fig. 9c). By western blotting we confirmed that α-syn was present in these exosomal fractions, along with autophagic protein LC3 I and P62/SQSTM1, whereas its levels appeared elevated in conditioned medium of PD-EXO treated BV2 cells (Fig. 9b). Collectively, not only did autophagy influences α-syn aggregation, but was also involved in the secretion of exosome-associated α-syn.

### BV2-produced exosomes contributes to α-syn aggregation via a hostile microenvironment in SH-SY5Y cells

Numerous studies have demonstrated that increased microglial activation and subsequent neuroinflammation contribute to neurotoxicity in PD^42,43^. On the other hand, α-syn contributes to the disruption of cellular function, which suggests a central role that α-syn plays in neurotoxicity of PD^2,56–61^. In order to study the effect of extracellular α-syn and inflammatory cytokines to the microenvironment, we exposed SH-SY5Y cells to exosomes prepared from conditioned mediums of PD-EXO-treated and PBS-treated BV2 cells. Nevertheless, no significant changes were observed in the cell viability (Figure not included). Interestingly, in these PD-EXO-treated conditions, a significantly higher level of the aggregated form of α-syn, in comparison with that observed in SH-SY5Y cells cultured with control exosomes, was detected (Fig. 9d). This was paralleled by a marked increase of phosphorylated α-syn expression in the SH-SY5Y cells after exposed to exosomes derived from PD-EXO-treated BV2 cells. No significant changes were observed for monomeric α-syn.

## Discussion

One of the major challenges in PD research is the lack of reliable and practical biomarkers that serve for early diagnosis and prognosis. In this study, we initially purified exosomes from the plasma, derived from PD patients and healthy controls, and subsequently investigated the changes of plasma exosomal α-syn levels and identified the α-syn species involved in PD progression. Consistent with previous studies^13^, our results show an elevation of exosomal total α-syn in plasma in PD patients compared to healthy controls. Similarly, a higher level of exosomal α-syn oligomers, rather than fibrils, was detected in PD patients. Findings from the seeding capacities and neurotoxicity of different α-syn species confirmed the pivotal role of α-syn oligomeric species in PD progression^2,60^. Given that exosomes facilitate the communication between the brain and the circulatory system, the identification of alteration in plasma exosomal α-syn oligomers could possibly be developed as a potential biomarker. The biogenesis and cargo loading of plasma α-syn-associated exosomes remain to be investigated as part of future research.

In the next section, plasma exosomes derived from PD patients were stereotaxically injected into the striatum of mice brains as a single dose. Fluorescence labeling revealed that plasma α-syn-associated exosomes could spread throughout the brain, indicating the transmission capacity of peripheral exosomes. Here, we demonstrate an obvious variance in the uptake rate of α-syn-carrying exosomes in various brain cell types. Specifically, plasma α-syn-associated exosomes were uptaken by microglia cells with no apparent internalization to astrocytes and neurons. A great quantity of PKH26-labeled exosomes was internalized into BV2 mouse microglia and HM cell lines, when these cells were co-cultured with exosomes. The underlying mechanism of such variance remains unclear. Furthermore, the presence of increased and reactive microglia suggests that these cells may be activated owing to phagocytic abnormalities in vivo. Consistent with the in vivo experiments, microglial cells efficiently took up plasma exosomes derived from PD patients, leading to increased expression of microglial marker protein IBA1, increased proliferation, and exhibiting elevated secretion of the proinflammatory cytokines and nitric oxide. Increasing evidence has shown that microglia-associated neuroinflammation may contribute to this neurotoxicity by secreting inflammatory mediators^32^. Elucidating the ligand–receptor interaction on the microglial surface could possibly reveal the role of microglia in the recognition and presentation of exogenous exosomes. In fact, it has been reported that toll-like receptors (TLRs), especially TLR2 and TLR4 mediates α-syn-induced activation of microglia^30,31,62–64^. Specifically, a recent study has demonstrated that neuron-released α-syn is an endogenous agonist for microglial TLR2 through which microglia are activated and become neurotoxic^30^. Specifically, our results were consistent with previous studies showing that partial α-syn located to the surface of the exosomes^10^. Another major study of tauopathies focuses on the fact that microglial receptor CX3CR1 plays a key role in the internalization of tau, a progress that also been indicated to been crucial in the synucleinopathy pathogenesis^65^. Besides, it has been reported that exosomes can be taken up via endocytosis^66^. Hence, whether these α-syn-binding partners contribute to exosome-mediated microglial activation and inflammatory response requires further study.

Although much attention has been paid to the intracellular processes of a-syn synthesis and degradation, little is known about the factors that regulate the extracellular protein levels. In this study, dynamic and live cell imaging indicated efficient uptake of extracellular exosomes and trafficking to the lysosomes that may result in an imbalance state of lysosomal degradation. On the other hand, phagocytosis of plasma exosomes derived from PD patients led to the accumulation of P62 and downregulated expression of LC3 II and Beclin1 in BV2 cells, indicating a blockage of autophagy. The AKT/mTOR pathway is a classical signaling pathway in regulating autophagy. We found that the AKT/mTOR pathway was involved in microglia autophagy inhibition that induced by plasma exosomes from PD patients, consistent with the study that activation of TLR2 resulted in the accumulation of a-syn aggregates in neurons as a result of inhibition of autophagic activity through regulation of the AKT/mTOR pathway^67^. It is widely believed that dysfunction of autophagy–lysosomal pathway appears in PD pathological process, driving the abnormal degradation processes of α-syn. Our data suggested a difference in both intracellular α-syn accumulation and exosomal α-syn secretion into extracellular cell supernatants between experimental groups and normal groups. Consistent with this, recent studies have revealed a strong correlation existing between the autophagy–lysosomal pathway and exosomal secretion^11,27,28^. To achieve the dynamic balance of intracellular membranous compartments, endosomal cargo could be transported to various cellular destinations, such as lysosomes, Golgi apparatus, and plasma membranes where exosomal biogenesis occurs. Studies have also suggested that autophagy–lysosomal pathway cooperated with exosomal secretion in clearing toxic intracellular proteins^28^. Taken together, exosomes could act as an efficient scavenger of eliminating aggregated proteins to compensate effects of autophagy–lysosomal dysfunction in the neurodegenerative diseases^68,69^. This mechanism is supported by data showing that inhibition of the autophagy–lysosome pathway can trigger the secretion of exosome-mediated α-syn in vitro^11,27,28,70^. These analyses showed that the interaction between exosome biogenesis and cellular degradation pathways might involve the pathological protein aggregation and secretion.

The biological function of microglia in the propagation of α-syn is poorly understood. Our data provide evidence that BV2 cells internalize the pathological exosomal α-syn and secrete α-syn via exosomes, which efficiently transmit to SH-SY5Y cells. Notably, immunofluorescence of human-specific α-syn from brains of exosome-injection models showed that exogenous α-syn is localized within neurons. In addition, we demonstrated that exogenous α-syn was taken up by neurons in the striatum and can be transferred to the cortex and SNpc over 2 weeks. Finally, activated microglia are sufficient to drive α-syn pathology and correlate with the spread of pathological α-syn in the brain. This suggests that the communication between microglia and neurons might be exemplified by the transport of exosomal cargo. The interventions to deplete microglial activation and to inhibit exosomal synthesis require further research.

In summary, our study has revealed significant implications regard to the crucial role of microglia and exosomes in a-syn propagation. Moreover, exosomes with toxic a-syn can activate microglia cells, thereby inhibiting autophagy activity, reducing the scavenging efficiency, and further promoting the accumulation and transmission of a-syn.

## Supplementary information

Video
